# Combined EMD-sLORETA Analysis of EEG Data Collected during a Contour Integration Task

**DOI:** 10.1371/journal.pone.0167957

**Published:** 2016-12-09

**Authors:** Karema Al-Subari, Saad Al-Baddai, Ana Maria Tomé, Gregor Volberg, Bernd Ludwig, Elmar W. Lang

**Affiliations:** 1 Department of Biology, Institute of Biophysics, University of Regensburg, Regensburg, Germany; 2 Department of Linguistics, Literature and Culture, Institute of Information Science, University of Regensburg, Regensburg, Germany; 3 Department of Electrical Engineering, Telecommunication and Informatics, Institut of Electrical Engineering and Electronics, Universidade de Aveiro, Aveiro, Portugal; 4 Department of Psychology, Pedagogics and Sport, Institute of Experimental Psychology, University of Regensburg, Regensburg, Germany; University of Minnesota, UNITED STATES

## Abstract

Lately, Ensemble Empirical Mode Decomposition (EEMD) techniques receive growing interest in biomedical data analysis. Event-Related Modes (ERMs) represent features extracted by an EEMD from electroencephalographic (EEG) recordings. We present a new approach for source localization of EEG data based on combining ERMs with inverse models. As the first step, 64 channel EEG recordings are pooled according to six brain areas and decomposed, by applying an EEMD, into their underlying ERMs. Then, based upon the problem at hand, the most closely related ERM, in terms of frequency and amplitude, is combined with inverse modeling techniques for source localization. More specifically, the standardized low resolution brain electromagnetic tomography (sLORETA) procedure is employed in this work. Accuracy and robustness of the results indicate that this approach deems highly promising in source localization techniques for EEG data.

## 1 Introduction

During the last decades, functional imaging techniques like functional magnetic resonance imaging (fMRI) and positron emission tomography (PET) dominated in neuroscientific research. Concomitantly, the importance of the technically much simpler, but less straightforward to analyze, electroencephalography (EEG) declined to some degree. Still, EEG plays an important role thanks to its high temporal resolution in the millisecond range and its direct access to neuronal activation rather than measuring it indirectly via the BOLD effect as in fMRI. Brain source imaging and reconstruction from continuous and single-trial EEG/MEG data thus have received increased attention to improve our understanding of rapidly changing brain dynamics, and using this information for improved real-time brain monitoring, brain computer interfaceing (BCI), and neurofeedback [1]. Recently, several new beamformers have been introduced for reconstruction and localization of neural sources from EEG and MEG. Beamformers provide a versatile form of spatial filtering suitable for processing data from an array of sensors [2].

Thus EEG provides dynamic information on submillisecond time scales which can be combined favorably with fMRI measurements which provide complementary high resolution information on small spatial scales in the millimeter range [3–7]. EEG reflects voltages generated mostly by excitatory postsynaptic potentials (EPSPs) from apical dendrites of massively synchronized neocortical pyramidal cells. Ionic current inflow at dendritic synapses and ionic outflow at the soma induce current dipoles at the pyramidal cells which finally cause the event-related membrane potentials (ERPs) seen in EEG recordings. Unfortunately, these source imaging techniques [8, 9] face the problem of ambiguity of the underlying static electromagnetic inverse problem. That is to say, the signals measured on the scalp surface do not directly indicate the location of the active neurons in the brain. Many different source configurations can generate the same distribution of potentials and magnetic fields on the scalp [10, 11]. Thus, the analysis of such EEG data is quite involved, encompassing machine learning and signal processing techniques like feature extraction [4, 12] and inverse modeling [13]. For timely accounts of recent advancements and actual challenges in dynamic functional neuroimaging techniques, including electrophysiological source imaging, multimodal neuroimaging integrating fMRI with EEG/MEG, and functional connectivity imaging see the reviews of Bin He [14] and Jatoi et al. [15]. Additionally, a systems level approach to understanding information processing in the human brain is offered by Edelman et al. [16] who advocate substantial efforts to shape the future of systems neuroengineering. Furthermore, for a recent open source toolbox, named *Brainstorm*, which offers tools to analyze MEG/EEG data, combine it with anatomical MRI data and locate underlying neuronal sources of activation, see Tadel et al. [17].

Source localization affords solving an inverse problem in EEG source analysis which is highly ill-posed due to a large *p*, small *n* problem setting [18]. Unique solutions can, however, be achieved by imposing additional constraints to the resulting optimization problem. Such constraints are often of a purely mathematical nature, but biophysically realistic constraints have been formulated as well (see for example LAURA [19]), [20, 21]. Source localization methods use measured scalp potentials in the microvolt range, and apply signal processing techniques to estimate current sources inside the brain which best explain the observations. The analysis first predicts scalp potentials resulting from a hypothetical current distribution inside the head—this is called the *forward problem*[22–25]. In a second step, these simulations are used in conjunction with the electrode potentials measured at a finite number of locations on the scalp to estimate the current dipole sources that fit these measurements—this is called the *inverse problem*[8, 13]. Over the years, researchers have developed non-parametric (also referred to as distributed source models or source imaging) as well as parametric (also called equivalent current dipole methods or spatio-temporal dipole fit models) approaches to tackle the source localization problem [13, 26]. Source localization accuracy depends on several factors like head-modeling errors [27, 28], source-modeling errors and measurement noise contributions [29]. Also it has been pointed out that the scalp potential needs to be sampled with electrodes evenly and densely distributed along the scalp surface [30]. Localization accuracy increases in a non-linear fashion with the number of electrodes, and estimates indicate that probably no less than 500 electrodes would be needed for an accurate sampling of the surface potential distribution [31, 32]. But it has also been pointed out recently that the absolute improvement in accuracy decreases with the number of electrodes [33]. Bayesian approaches, have been reviewed recently [34], allow to compare several models and indicate that spatial localization precision in the milimeter range can be achieved reliably. Localization accuracy increases in a non-linear fashion with the number of electrodes, and the latter need to spread over all the scalp surface homogeneously. If electrodes are concentrated in certain scalp segments, source localization can turn awfully wrong [35]. For practical purposes, Baillet et al. [36, 37] suggested a spatial accuracy of 5 [*mm*] and a temporal accuracy of 5 [*ms*], respectively. Among the many source localization methods available, low resolution electrical tomography (LORETA) [38] and its extensions standardized LORETA (sLORETA) [39] and exact LORETA (eLORETA) [40, 41] are the most commonly employed techniques. Especially sLORETA seems to outperform other techniques in most practical situations. Hence it is considered the method of choice in this study.

Source localization is usually applied to the original signals (scalp potentials) collected at the various electrodes. These original signals consist of *N*_*e*_ non-stationary time series of potential fluctuations at specified scalp locations, with *N*_*e*_ < 100 in most practical applications. These time series can be collected in a data matrix **Y** of dimension *N*_*e*_ × *N*_*T*_ with *N*_*e*_ ≪ *N*_*T*_ and the *N*_*T*_ the number of time samples. But besides source localization, feature extraction and classification represents another major data analysis objective for unravelling the information buried in such brain signals. Powerful supervised as well as unsupervised machine learning techniques are available for characterizing the recorded potential fluctuations subject to predefined constraints imposed during the analysis process. Recently, an informed decomposition approach, which built upon constrained optimization approaches [42] for independent components analysis, has been proposed to better model and separate distinct subspaces within EEG data [43]. While supervised techniques require expert knowledge, unsupervised methods such as exploratory matrix factorization (EMF) methods, variously known as blind source or signal separation (BSS) techniques [44] or empirical mode decomposition (EMD) methods [12, 45, 46] offer versatile tools for transforming the registered signals into more elusive and informative representations. It is one of the objectives of this study to investigate the specific advantages of applying such methods as preprocessing techniques, and applying source localization to the modes extracted from such methods instead of to the raw signals themselves.

Although ICA has been applied successfully to EEG data sets (see for example [47]), because of the inherently non-stationary nature of recorded EEG signals, EMD and its extension called ensemble EMD (EEMD) [46], is favored in this investigation over EMF methods like principal (PCA) or independent (ICA) component analysis which require at least wide-sense stationary signals. EMD utilizes an empirical knowledge of intrinsic oscillations of a time series in order to represent the latter as a superposition of oscillatory components with instantaneous frequencies derived from their time-dependent phases. EMD thus adaptively and locally decomposes any non-stationary signal into a sum of intrinsic mode functions (IMFs) which represent zero-mean, amplitude- and (spatial-) frequency-modulated components, henceforth called modes. IMFs are referred to as event-related modes (ERMs) in case of a decomposition of event-related potentials (ERPs), i. e. averages over many trials, of EEG data [4]. In a recent comparative study we analyzed combined EEG/fMRI data sets with with a combination of EEMD and ICA techniques. The raw data have first been decomposed into intrinsic modes by EEMD, yielding stationary components which then have been further analyzed by an ICA [6]. Another recent investigation combined ICA with EEMD by using an interesting ERM as reference for a constrained ICA (cICA or ICA-R) [48]. There it was shown that ICA with reference indeed extracts an independent mode which is very similar to the corresponding intrinsic mode which was taken as reference signal. The latter corroborates that ICA some of the independent components are indeed very similar to intrinsic modes extracted by EMD.

The experimental paradigm used in our previous study [3, 4] was a contour integration task, applied to a group of 19 probands and 300 trials each. A large set of Gabor stimuli was presented repeatedly which occasionally contained a contour made up by a subset of collinearly oriented Gabor patches. The participants had to signal the preception of contour or non-contour stimuli with a manual response.

In our recent study [4], brain electrodes have been distinguished according to the timing of their stimulus response. Early responses were recorded at electrodes localized in the occipital and parietal areas of the brain, while late responses were located in frontal and medio-temporal areas of the brain. Early and late responses, manifested in ERP components *P*100 and *N*200, turned out most discriminative in detecting significant response differences to contour and non-contour stimuli. These components represent the first and second prominent ERP peaks with latencies of roughly 100 [*ms*] and *N*200 [*ms*] after stimulus onset. It has been shown in [4] that the event-related modes ERM5 most closely reflected the dominant oscillation of the grand average EEGs of the various subjects.

In this study, we propose, for the first time, to combine an EEMD analysis with a source localization scheme, more specifically an sLORETA source estimation. We investigate whether an EEMD analysis can provide underlying characteristic modes which, when fed into an sLORETA analysis, can help to localize sources of neuronal activity reflecting cognitive processing during the contour integration task performed in our recent study [4], employing CT (contour true) and NCT (non-contour true) stimuli. Hence, measured EEG responses are subjected to mode decomposition techniques, more specifically to an EEMD, and sLORETA is applied to the event—related modes (ERMs) extracted to solve the source imaging problem. Note that, contrary to our recent study, no channel pooling is applied in this study to avoid any adverse effect on localization accuracy. Although a wealth of source localization procedures meanwhile exist [9, 13–15, 34], citeEdelman15, we considered sLORETA because of its straightforward implementation and its good performance in real applications [41].

## 2 Materials and Methods

### 2.1 EEG Data

The data used in this study are EEG recordings collected during a contour integration task [4, 12]. The data was collected from 64 electrodes (BrainAmp MR plus, Brain Products, Gilching, Germany) placed according to the 10 − 10 system. 62 electrodes were used to record scalp EEG potentials, and were referenced against the FCz electrode during recording. EEG signals were sampled at 5 [*kHz*] (later reduced to 500 [*Hz*]). Eye movement artifacts have been monitored by an electrode located below the left eye (electrooculogram, or EOG). To simplify the off-line removal of cardioballistic artifacts, an electrocardiogram (ECG) electrode was placed below the left scapula.

**Fig 1:**
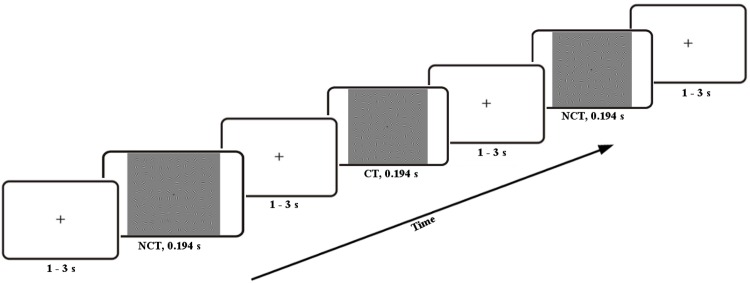
Stimulus protocol including Gabor patches either forming a contour (CT) or none (NCT).

The study encompassed 18 subjects who participated in the study, 5 male and 13 female with an age varying between 20 and 29, and an average of (22.79 ± 2.7) [*years*]. Note that one subject is omitted due to an error when saving data. During the experiment, subjects were seated in a sound-attenuated chamber in front of a monitor while applying two visual Gabor stimulus conditions, i. e. *contour true* (CT) and *non-contour true* (NCT). Each of a these visual stimuli was presented for 194 [*ms*], followed by a blank screen after a random interval from 1 − 3 [*s*] (see Fig 1). Then the next trial started after having received the response of the proband or after a time-out of 3 [*s*] in case the subject did not respond. This EEG data [4, 49] was recorded jointly with fMRI data as described in [3, 49].

The study was approved by the ethics committee of the University of Regensburg (reference number 10 − 101 − 0035). All participants provided their written informed consent about their participation in the study. All subjects were subjected to a procedure in accord with the principles laid down in the Helsinki declaration. This procedure was approved by the ethics committee of the University of Regensburg.

### 2.2 Ensemble Empirical Mode Decomposition

Empirical Mode Decomposition (EMD) represents an adaptive data analysis tool for non-linear and non-stationary time series. It has been proposed by [45]. EMD is a method of breaking down a signal into components known as Intrinsic Mode Functions (IMFs). The latter locally represent pure oscillations which reflect characteristic time scales of the data and shall satisfy only the following requirements [45]:

- The number of extrema and zero crossings must be equal or differ by a maximum of one.
- The mean value of the envelope defined by the local maxima and the envelope defined by the local minima must equal zero at any point.

The process of extracting IMFs is called *sifting*. The EMD algorithm for decomposing the original signal *x*(*t*) into intrinsic modes can be summarized as follows:

1. Identify the extrema (both maxima and minima) of the signal *ϕ*_*k*_(*t*) registered at the *k*-th electrode.
2. Construct the upper and lower envelopes *env*_*max*_(*t*) and *env*_*min*_(*t*) using a cubic spline interpolation scheme.
3. Calculate the mean of the two envelopes as $m\left(t\right)=\frac{\left[env_{max}\left(t\right)+env_{min}\left(t\right)\right]}{2}$
4. Subtract the mean value from the signal *h*(*t*) = *ϕ*_*k*_(*t*) − *m*(*t*)
5. Determine whether *h*(*t*) is an IMF or not by checking the two conditions as described above.
6. If *h*(*t*) is IMF, set *c*_*j*_(*t*) = *h*(*t*) and find the *j*+1—st IMF after updating $r\left(t\right)=\phi_{k}\left(t\right)-\sum_{j<(j+1)}c_{j}\left(t\right)$. Otherwise, update *ϕ*_*k*_(*t*) = *h*(*t*) and repeat steps 1 to 5.

The above sifting procedure will be repeated until all IMFs have been extracted. At the end of the decomposition, the original signal can be represented by an expansion into its underlying modes plus a non-oscillating residuum:

$$\begin{array}{c} \phi_{k}\left(t\right)=\sum_{j=1}^{J}c_{j}\left(t\right)+r\left(t\right) \end{array}$$ (1)

where *J* denotes the number of IMFs, the *c*_*j*_(*t*) represent the IMFs and *r*(*t*) represents the remaining non-oscillating trend.

EMD provides a useful method for analyzing natural signals, which are most often non-linear and non-stationary. Consequently, its application deems appropriate for an EEG analysis. The EMD is a data-driven method which is completely unsupervised and does not need to obey additional constraints like competing exploratory data decomposition techniques. In addition, EMD assures the perfect reconstruction property, i. e. the sum of all the extracted IMFs with the residual trend yields the original signal without information loss or distortion. This also implies that component amplitudes do not suffer from any scaling indeterminacy. However, one of the major shortcomings of plain EMD, when applied to real signals, is the frequent appearance of mode mixing. It is a consequence of signal intermittency. To alleviate this problem, Wu et al. [46] proposed a noise-assisted variant called Ensemble Empirical Mode Decomposition (EEMD). It is based on studies of the statistical properties of fractional Gaussian noise [50, 51]. These studies showed that EMD can be considered an adaptive dyadic filter bank when applied to fractional Gaussian noise. EEMD is based on repeatedly adding white noise to the target signal while applying EMD

$$\begin{array}{c} \phi_{k}\left(t\right)={\tilde{\phi }}_{k}\left(t\right)+\epsilon_{n}\left(t\right)=\sum_{j}c_{n}^{\left(j\right)}\left(t\right)+r_{n}\left(t\right), \end{array}$$ (2)

where ${\tilde{\phi }}_{k}\left(t\right)$ is the true, noiseless signal, *ϵ*_*n*_(*t*) is the white noise and $c_{n}^{\left(j\right)}=c^{\left(j\right)}+\epsilon_{n}\left(t\right)$ represents the IMF obtained for the *n*-th noise observation. These IMFs are estimated as an ensemble average which suppresses noise contributions due to self-averaging of the latter.

### 2.3 Source Localization

Our recent EEMD analysis [4] of the EEG data mentioned above revealed underlying event-related modes (ERMs) which showed clear differences between stimulus modalities and exhibited a time delay (≈ 70[*ms*]) when the modes’ contributions to frontal versus occipital electrodes were considered. This suggests that a study of the related source localization problem might reveal spatio-temporal features not obtainable from a corresponding source localization study of the raw EEG signals.

Electrophysiological source imaging (ESI) [14, 52] is the scientific field allocated to modeling and evaluating the spatiotemporal dynamics of neuronal currents throughout the brain that generate the electric potentials and magnetic fields measured with electromagnetic (EM) recording technologies [53]. Thus, over the past few decades, localizing electrical sources in the brain from surface recordings has attracted the attention of many EEG/MEG researchers.

The EEG neuroimaging problem actually consists of a forward and an inverse modeling problem. With forward modeling [54, 55], one is interested in predicting the expected potential distribution on the scalp from given intracranial activities which is frequently modeled as electric current dipole sources **d**_*i*_ ∝ ∇ ⋅ **j**(**r**_*i*_), *i* = 1, …, *N*_*v*_ with **j**(**r**_*i*_)[*A*/*m*^2^] the current density and ∇ the nabla operator calculating the divergence of the ionic currents. By invoking Ohm’s law, the Poisson equation can be derived which relates the scalp potentials to the current density distribution inside the brain. Thus, in practice, forward modeling amounts to predicting the set of electric potentials {**Φ**(**r**_*k*_,**d**_*i*_)|*k* = 1, …, *N*_*e*_, *i* = 1, …, *N*_*v*_} which would be measured at any scalp electrode *k* if some current dipole sources **d**_*i*_ were active inside the brain at discrete locations **r**_*i*_. The inverse problem can be defined as the problem of estimating the current density **j**(**r**_*i*_), more precisely its equivalent current dipole moments **d**_*i*_, that generated the measured electrical potential. Whereas the general forward solution is well-defined, the inverse solution is ill-posed because of the large number *N*_*v*_ of parameters, i. e. current dipole moments **d**_*i*_ estimated at locations **r**_*i*_ compared with the small number *N*_*e*_ ≪ *N*_*v*_ of observations, i. e. measured scalp potentials *ϕ*(**r**_*k*_). Thus some regularization is needed which introduces additional constraints to assure a unique solution to the related optimization problem. Over the years, a number of non-parametric as well as parametric techniques [13] have been developed. One of the most robust methods for source localization is referred to as standardized low resolution brain electromagnetic tomography (sLORETA) which was introduced by [39]. For single point sources and noiseless data, sLORETA has been shown to provide an exact source localization even for blurred images. However it was shown also that the precision with which sources can be localized strongly depends on the number, and even more so on an even distribution over the scalp surface, of electrodes from which electrical potentials are collected [35].

#### 2.3.1 sLORETA

The forward problem amounts to solving Poisson’s equation

$$\begin{array}{c} \nabla^{2}\phi \left(r_{k},t\right)=-\epsilon^{-1}\rho_{q}\left(r,t\right) \end{array}$$ (3)

for the electrical potential *Φ*(**r**_*k*_, *t*), registered at scalp location **r**_*k*_ at sampling time *t*, as function of the charge density *ρ*_*q*_(**r**, *t*) inside the brain. Biophysically, scalp potentials can be described as stemming from ionic currents in apical dendritic trees of pyramidal neurons resembling dipolar charge distributions at locations **r**_*i*_ and having dipole polarization **d**_*i*_. According to the superposition approximation, the total potential at any scalp electrode location **r**_*k*_ amounts to

$$\begin{array}{ccc} \phi (r_{k},t) & = & \sum_{i}\phi (r_{k},d_{i}\left(r_{i},t\right)) \end{array}$$ (4)

$$\begin{array}{ccc} & ⋍ & \sum_{i}g(r_{k},r_{i})\cdot d_{i}\left(t\right) \end{array}$$ (5)

where **g**(…) is called the gain or lead field which depends on dynamic electric susceptibilities inside the brain. Given *N*_*e*_ electrodes, *N*_*v*_ dipoles and *T* discrete time samples, the measured scalp potentials at all *N*_*e*_ electrode locations at times *t*_1_, …, *t*_*T*_ can be collected into an *N*_*e*_ × *T*—dimensional data matrix **Φ**(*t*) which is estimated via

$$\begin{array}{c} O_{c}\Phi \left(t\right)=O_{c}G\left(r_{j},r_{i}\right)D\left(r_{i},t\right)+E_{n}\left(t\right) \end{array}$$ (6)

Note that all EEG signal-related quantities, i. e. **Φ**, **G**, are conveniently re-referenced to an average EEG signal by applying the *N*_*e*_ × *N*_*e*_—dimensional centering operator **O**_*c*_ = **I** − **1** **1**^*T*^(**1**^*T*^ **1**)^−1^ which obeys the relation **O**_*c*_ **1** = **0**. Note that source localization does not depend on the choice of the reference electrode, as long as the reference is correctly integrated into the model [35]. Further, **G** represents the *N*_*e*_ × *N*_*v*_—dimensional gain or lead field matrix, **D**(**r**_*i*_, *t*) the *N*_*v*_ × *T*—dimensional matrix of current dipole moments **d**_*i*_(*t*_*n*_) ≡ **d**(**r**_*i*_, *t*_*n*_) = (*d*_*x*, *i*_(*t*_*n*_), *d*_*y*, *i*_(*t*_*n*_), *d*_*z*, *i*_(*t*_*n*_))^*T*^ at a finite set $I=\{i|1,\ldots ,N_{v}\}$ of grid points **r**_*i*_ and a finite set of discrete time points *t* = *t*_1_, …, *t*_*T*_, and **E**_*n*_ denotes additive noise. The *N*_*v*_ grid points are located in cortical gray matter and the hippocampus. While the gain matrix **G** is estimated via solving the forward problem [23, 54, 56], the inverse problem tries to deduce the dipole matrix **D** from electrical potentials **Φ** measured at electrode locations **r**_*k*_ at any discrete time *t*_*n*_.

Non-parametric optimization methods solve the inverse problem by estimating the dipole matrix D* which maximizes the posterior probability distribution *p*(**D**|**Φ**) of current dipole sources **d**_*i*_(*t*_*n*_) given the observations **Φ**(**r**_*k*_, *t*_*n*_). Assuming a Gaussian posterior density, the corresponding log-posterior density is related to an energy functional *F*_*α*_(**d**) = **R**(**d**) − *α* **L**(**d**), which consists of the data log-likelihood representing a reconstruction error **R** = ∥**Φ** − **G** **D**∥^2^ and a log-prior which constitutes a regularization term [57]. In case of sLORETA, the latter is, in the spirit of Tikhonov regularization, taken as **L**(**D**) = ∥ **D**∥^2^ yielding a minimum norm least squares estimate

$$\begin{array}{c} D_{MNE}=G{\left(GG^{T}+\alpha O_{c}\right)}^{†}\Phi . \end{array}$$ (7)

The latter becomes standardized by the square root of its *N*_*v*_ × *N*_*v*_—dimensional co-variance matrix **Σ**_*D*_ = **G**^*T*^(**G** **G**^*T*^+*α* **O**_*c*_)^†^ **G**. Thus, at any given time point *t*, the (3 × 1)—dimensional vector of the estimated standardized dipole moment $\tilde{d}$ at voxel location **r**_*i*_ is obtained as [41, 58]

$$\begin{array}{c} dMNE,i\left(t\right)\equiv d_{MNE}\left(r_{i},t\right)={\left[\Sigma_{D}\right]}_{ii}^{-1/2}d\left(r_{i},t\right) \end{array}$$ (8)

Finally the sLORETA brain maps result from computing estimates of the equivalent standardized current dipole energy at all grid points **r**_*i*_.*i* = 1, …, *N*_*v*_

$$\begin{array}{c} E_{dip}\left(r_{i}\right)⋍d_{MNE,i}^{T}{\left({\left[\Sigma_{D}\right]}_{ii}\right)}^{-1}d_{MNE,i} \end{array}$$ (9)

where **d**_*MNE*, *i*_ is the minimum norm current dipole moment estimate at the *i*-th voxel and [**Σ**_*D*_]_*ii*_ is the (3 × 3)—dimensional *i*-th diagonal block of the co-variance matrix **Σ**_*D*_ [13, 39, 41].

Note that because pyramidal neurons span all cortical layers, the model is often simplified by assuming that, at each grid point, the direction of the ionic currents inside the apical dendritic trees, and thus the equivalent dipole moment orientation, is orthogonal to the surface. Then only its amplitude needs to be estimated. In that case, the matrix **D** has dimension *N*_*v*_ × 1 and each *i*—th element corresponds to the amplitude of the *i*—th voxel, and the dimension of the gain matrix, as well as $\Sigma_{\hat{D}}$, also changes to *N*_*e*_ × *N*_*v*_.

### 2.4 Data analysis

The EEG data were processed using the EEGLAB toolbox [59] and the recently integrated EMDLAB toolbox [12], before the data were analyzed by sLORETA. EEG artifacts (i. e. eye blink and eye movements, heart beat and muscle noise) were removed by independent component analysis (ICA) [60].

The ERPs were analyzed by the sLORETA software [39] available at (http://www.uzh.ch/keyinst/loreta.htm) to estimate equivalent current source density dipole moments. Briefly, sLORETA calculates the standardized source current dipole moments at each of the 6239 voxels located in the gray matter and the hippocampus of the MNI-reference brain. This calculation is based upon a linear weighted sum of the scalp electric potentials. sLORETA estimates the underlying sources under the assumption that the neighboring voxels should have a maximally similar electrical activity. Source current dipole moments in each voxel were compared between the two stimulus conditions a paired t-test. For this comparison, sLORETA software performs a *non-parametric randomization* of the data [61].

## 3 Results

The following section will present results obtained from a combined EEMD-sLORETA analysis of EEG recordings from 18 subjects during a contour integration task. This EEG data has been recorded simultaneously with fMRI scans.

**Fig 2:**
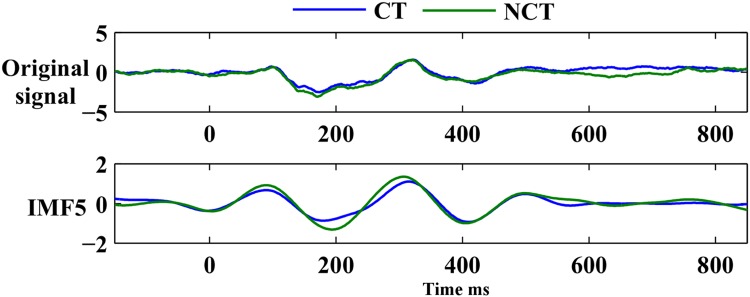
Comparison of the original EEG recording (grand average over 18 subjects of channel O2) with ERM 5.

**Fig 3:**
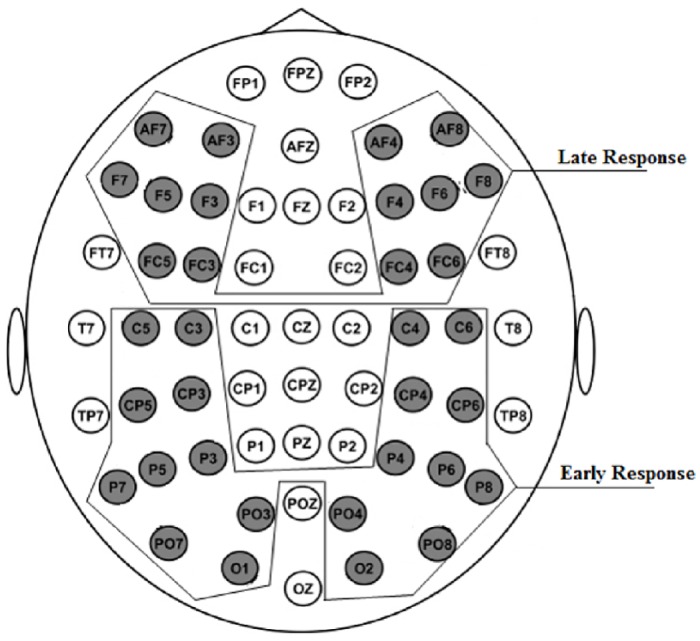
Electrode placement according to the 10—20 system, and pooling into early and late response signals.

Results concerning the EEMD analysis of this EEG data has been published recently by [4]. In this study, the results related to raw data are presented at the level of event-related potentials (ERPs). Raw data is then decomposed with EEMD using single trial recordings. Based on the analysis of the previous study [4], source localization results obtained with raw data are compared with results obtained from the most informative event-related mode *ERM*5. As can be seen in Fig 2, the intrinsic mode ERM5 most closely reflects the prominent ERPs of the raw data set. The latter corresponds to a grand average over 18 subjects of the signals recorded at channel O2 (see Fig 3). Within each average time series, the two most prominent potentials of each ERM, denoted, according to their related ERPs and their latencies after stimulus onset, as *P*100 (positive response roughly 100[*ms*] after stimulus onset) and *N*200 (negative response roughly 200[*ms*] after stimulus onset), will be considered. These response amplitudes were most clearly seen in ERM5 and showed statistically significant differences in response to contour versus non-contour Gabor stimuli. The EEMD analysis further revealed a delay which amounts to 70[*ms*] when comparing response latencies at occipital and frontal brain areas. Early *P*100 and *N*200 responses occurred at electrodes located in the occipital, parietal and parieto-temporal areas of the brain, while late *P*100 and *N*200 responses appeared at electrodes located in frontal and fronto-temporal brain areas. The same potentials, when appearing at electrodes in central brain areas, showed bimodal early/late response signatures. Note that ERPs have been pooled as illustrated in Fig 3 (see [4]). Note further that the potentials *P*300 and *N*400 did not show any difference in latencies between early and late responses. A statistical paired T -test of differences in reconstructed response amplitudes to both stimulus conditions resulted in a series of paired T-test values. The latter served to compare, between the two stimulus conditions, *CT* and *NCT*, and for selected latencies, the response amplitudes which were reconstructed, employing sLORETA, from both the ERP and the intrinsic mode *ERM*5.

The current study is concerned with estimating the localization of the spatial sources related to these ERPs in the raw data as well as in the ERMs. For simplicity we confine our discussion to potentials appearing in mode *ERM*5. The inverse problem was solved by employing the sLORETA software package [39]. The related sLORETA values according to the Brodmann area (BA) per brain map are given for the sources identified.

### 3.1 Early Response

#### 3.1.1 ERP component P100 at 60-120 [ms])

**Fig 4:**
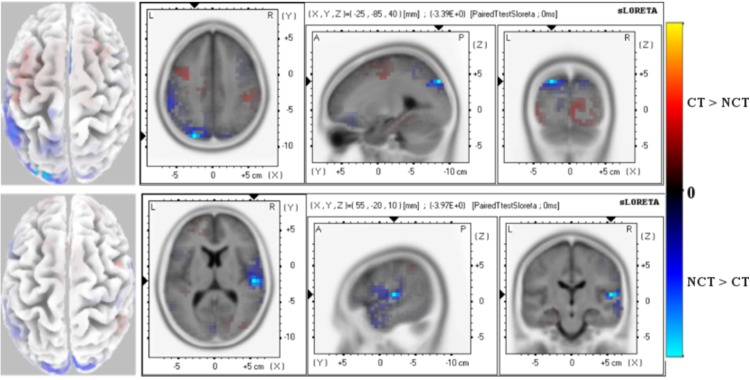
Early Response (60-120 ms) P100 ERP. Paired t-test values of significant potential amplitude differences at electrodes are illustrated at a significance level as specified. Views are axial, saggital and coronal. The left column shows the distribution on the scalp. All 62 electrodes were used as entries to the data matrix **Φ**. *(Top):* Raw ERP P100 with significance level *P* = 0.01. *(Bottom):* ERM5 extracted from the ERP P100 with significance level *P* = 0.001. Red color (positive paired T-test values) indicates that the *ERP* amplitude for the stimulus condition *CT* is larger than for condition *NCT* while blue color (negative paired T-test values) indicates that the *ERP* amplitude for the stimulus condition *NCT* is larger than for condition *CT*.

Fig 4 illustrates results of an sLORETA analysis of response differences in early stimulus responses for both stimulus conditions. Mean response amplitudes have been estimated for the interval 60 − 120 [*ms*] around the ERP *P*100 peak. Shown are significant paired t-test values for the differences, for both stimulus conditions, of potential readings from all 62 electrodes, as shown in Fig 3, have been used as entries to the data matrix **Φ**. The graphic illustrates significant differences for the raw ERP Fig 4-Top and the mode *ERM*5 Fig 4-Bottom. Blue and red colors thereby indicate negative or positive paired t-test values, respectively.

As can be seen in Fig 4-Top, differences of the raw ERP appear in the occipital and parietal brain areas of the left hemisphere at a significance level of *P* = 0.01. Also some weaker positive activity differences are detected in the temporal regions of the both hemispheres at significance level *P* = 0.05.

These results should be contrasted to those obtained from studying the mode *ERM*5 *P*100 of the EEMD analysis as it appears in the *ERP* potential. The most noticeable difference is that *ERM*5 shows highly localized, significant differences mainly in the temporal, occipital and parietal regions. There, the amplitude of the early *P*100 component of *ERM*5 is larger for the stimulus condition *NCT* than for condition *CT*. The highest differences appears in the temporal lobe at significance level *P* = 0.001.

Table 1 illustrates the significant differences results of the early *P*100 response of raw ERP and mode *ERM*5, respectively, in detail. The table summarize the Brodmann areas (BA), MNI coordinates and the neuroanatomical lobe of the voxels for the *P*100 early response that showed statistically the most significant differences of Brodmann area clusters.

**Table 1:** T-test statistics for early *P100 ERP* and *ERM5* response. The table shows coordinates of the most significant voxel of clusters. The sign of T-test values indicates the differences between stimuli (′−′*NCT* > *CT*, ′+′*CT* > *NCT*).

**ERP**						
X	Y	Z	T-value	Voxels-No	BA	Brain Lobe
−25	−85	40	−3.39	14	19*	Parietal
5	15	25	−3.17	8	24*	Limbic
**ERM5**						
X	Y	Z	T-value	Voxels-No	BA	Brain Lobe
55	−20	10	−3.97	21	(41**, 22*, 42*)	Temporal
10	−90	25	−3.15	79	(18*, 19*)	Occipital
−20	−80	35	−3.01	18	(19*, 40*)	Parietal

* *p* = 0.01

** *p* = 0.001.

#### 3.1.2 ERP component N200 at 150-210 [ms])

**Fig 5:**
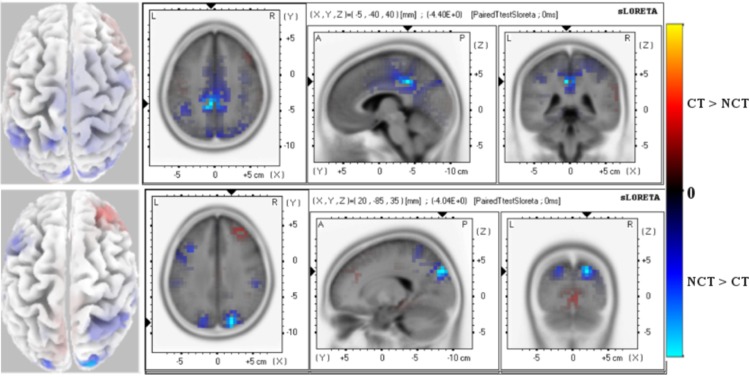
Early Response (150-210 ms) N200 ERP. Paired t-test values of significant potential amplitude differences at electrodes are illustrated at a significance level as specified. Views are axial, saggital and coronal. The left column shows the distribution on the scalp. All 62 electrodes were used as entries to the data matrix **Φ**. *(Top):* Raw ERP N200 with significance level *P* = 0.001. *(Bottom):* ERM5 extracted from the ERP N200 with significance level *P* = 0.001. Red color (positive paired T-test values) indicates that the *ERP* amplitude for the stimulus condition *CT* is larger than for condition *NCT* while blue color (negative paired T-test values) indicates that the *ERP* amplitude for the stimulus condition *NCT* is larger than for condition *CT*.

Next, Fig 5 illustrates paired t-test values for the *ERPN*200 as resulting from an analysis of the raw data Fig 5-Top and the mode *ERM*5 Fig 5-Bottom. Shown are significant differences in early stimulus responses. *N*200 early response differences of *ERPN*200 are mainly located in limbic lobe and parietal regions of the left hemisphere with significance level *P* = 0.001. There are also significant differences in the frontal and occipital regions at confidence level *P* = 0.01.

Comparing these results with the outcome of an analysis of mode *ERM*5, a much more focused significant difference in response activities to both stimulus conditions is located in the parietal and occipital cortexes of both hemispheres at a confidence level of *P* = 0.001. Some positive activity differences also show up in frontal areas of the right hemisphere where the amplitude of the early *N*200 component of *ERM*5 is larger for the stimulus condition *CT* than for condition *NCT*.

These results of early *N*200 response of raw ERP and mode *ERM*5 are summarized in Table 2. As can be seen from the table, all the results show a negative significant differences where the amplitude for the condition *NCT* is larger than for the condition *CT*. Early responses of raw ERP have been mainly observed for channels located in the limbic, parietal and frontal areas of the brain, while a highly significant early response of *ERM*5 has been observed for channels in the occipital, parietal and frontal areas of the brain.

**Table 2:** T-test statistics for early *N200 ERP* and *ERM5* response. The table shows coordinates of the most significant voxel of clusters. The sign of T-test values indicates the differences between stimuli (′−′*NCT* > *CT*, ′+′*CT* > *NCT*).

**ERP**						
X	Y	Z	T-value	Voxels-No	BA	Brain Lobe
−5	−40	40	−4.40	99	(31**, 24*)	Limbic
−15	−50	55	−4.35	127	(7**, 19*, 31*, 40*)	Parietal
−20	−45	50	−3.90	40	(5*, 31*)	Frontal
10	−60	30	−2.91	8	31*	Occipital
**ERM5**						
X	Y	Z	T-value	Voxels-No	BA	Brain Lobe
20	−85	35	−4.04	32	(7**, 19**)	Parietal
20	−90	35	−3.76	22	(7 *, 19 *)	Occipital
−40	10	40	−2.99	18	9 *	Frontal

* *p* = 0.01

** *p* = 0.001.

### 3.2 Late Response

#### 3.2.1 ERP component P100 at 120-180 [ms]

**Fig 6:**
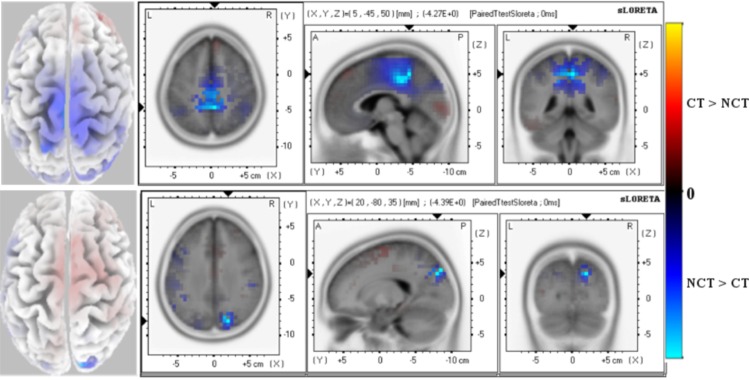
Late Response (120-180 ms) P100 ERP. Paired t-test values of significant potential amplitude differences at electrodes are illustrated at a significance level as specified. Views are axial, saggital and coronal. The left column shows the distribution on the scalp. All 62 electrodes were used as entries to the data matrix **Φ**. *(Top):* Raw ERP P100 with significance level *P* = 0.001. *(Bottom):* ERM5 extracted from the ERP P100 with significance level *P* = 0.001. Red color (positive paired T-test values) indicates that the *ERP* amplitude for the stimulus condition *CT* is larger than for condition *NCT* while blue color (negative paired T-test values) indicates that the *ERP* amplitude for the stimulus condition *NCT* is larger than for condition *CT*.

When it comes to consider late stimulus responses as seen in raw data sets (see Fig 6-Top), a *P*100 response peak appears delayed by 70 [*ms*]. Corresponding source activity differences between both stimulus modalities mainly show up in central areas. But if mode *ERM*5 is considered instead, highly focused activity differences appear (see Fig 6-Bottom). The significant activity differences are only seen in visual cortex of the right hemisphere. Again, mode *ERM*5 shows a much more focused activity distribution than the raw data set.

Table 3 summarizes, for late response of the *P*100 ERP, coordinates, T-values of the test statistics at different confidence levels, the Brodmann area and the anatomical area where the significant differences are. The highly significant differences are located at frontal and parietal regions (*P* = 0.001) while sub-lobar and limbic regions shows a significant differences of (*P* = 0.01).

**Table 3:** T-test statistics for late *P100 ERP* and *ERM5* response. The table shows coordinates of the most significant voxel of clusters. The sign of T-test values indicates the differences between stimuli (′−′*NCT* > *CT*, ′+′*CT* > *NCT*).

**ERP**						
X	Y	Z	T-value	Voxels-No	BA	Brain Lobe
5	−45	50	−4.27	199	(5**, 31**, 4*, 6*)	Frontal
5	−35	40	−4.21	146	(31**, 23*, 24*)	Limbic
5	−35	45	− 4.14	80	(7**, 4*, 31*, 40*)	Parietal
−45	−25	20	−2.97	9	13*	Sub-lobar
**ERM5**						
X	Y	Z	T-value	Voxels-No	BA	Brain Lobe
20	−80	35	−4.39	18	(7**, 19**)	Parietal
20	−80	30	−3.32	13	(7*, 19*)	Occipital

* *p* = 0.01

** *p* = 0.001.

When *ERM*5 is considered, significant results can be summarized in Table 3. As can be noted in the table, the significant results are focused in the occipital and parietal regions at significance level (*P* = 0.001).

#### 3.2.2 ERP component N200 at 200-260 [ms]

**Fig 7:**
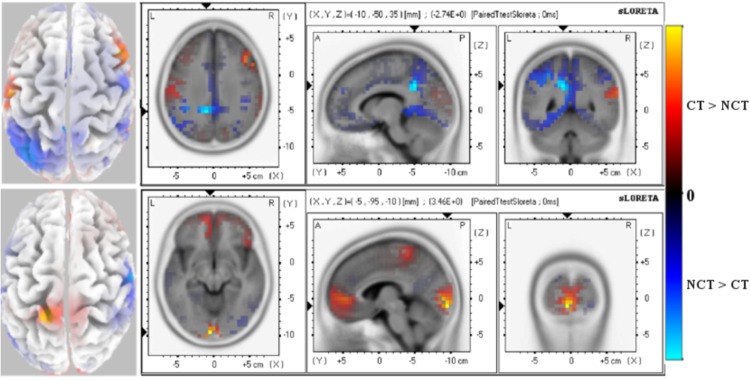
Late Response (200-260 ms) N200 ERP. Paired t-test values of significant potential amplitude differences at electrodes are illustrated at a significance level as specified. Views are axial, saggital and coronal. The left column shows the distribution on the scalp. All 62 electrodes were used as entries to the data matrix **Φ**. *(Top):* Raw ERP N200 with significance level *P* = 0.05. *(Bottom):* ERM5 extracted from the ERP N200 with significance level *P* = 0.01. Red color (positive paired T-test values) indicates that the *ERP* amplitude for the stimulus condition *CT* is larger than for condition *NCT* while blue color (negative paired T-test values) indicates that the *ERP* amplitude for the stimulus condition *NCT* is larger than for condition *CT*.

Considering the *ERP* *N*200 at the late response electrodes, significant activity differences show up in occipital and parietal regions of the left hemisphere with negative paired t-test values, but activity differences with slightly positive paired t-test values also appear in pre-frontal regions of the right hemisphere (see Fig 7-Top). Positive t-test values have been observed for channels located in the frontal areas of the brain, while negative t-test values has been observed for channels in the occipital and parietal area of the brain. Both positive and negative t-test values of the ERP are slightly differences at a significance level *P* = 0.05.

Again, if it comes to compare these results with those obtained by using only amplitudes of mode *ERM*5, highly focused significant activity differences are located in frontal and occipital areas with strongly positive paired t-test values while a clear focus of weakly negative paired t-test values also appears in parietal areas (see Fig 7-Bottom). These results are summarized in Table 4. As can be shown in the Table 4, positive differences of the conditions responses are located in the parietal and occipital regions of the brain while negative differences are detected in the frontal region.

**Table 4:** T-test statistics for late *N200 ERM5* response. The table shows coordinates of the most significant voxel of clusters. The sign of T-test values indicates the differences between stimuli (′−′*NCT* > *CT*, ′+′*CT* > *NCT*).

ERM5						
X	Y	Z	T-value	Voxels-No	BA	Brain Lobe
60	−10	45	−3.18	69	(4*, 6*, 10*, 45*, 47*)	Frontal
−5	−95	−10	3.46	59	(17*, 18*)	Occipital
−20	−40	70	3.21	6	3*	Parietal
−10	50	0	3.14	13	(10*, 32*)	Limbic

* *p* = 0.01.

**Fig 8:**
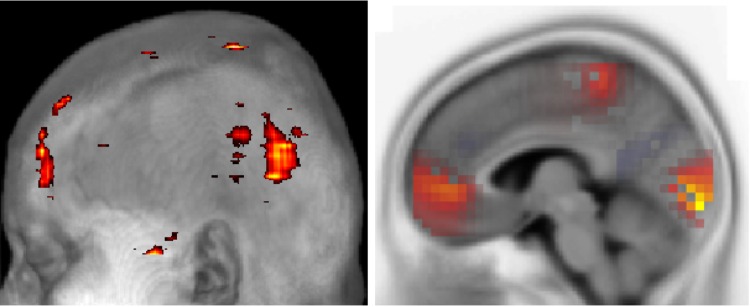
Saggital view of *left:* the intrinsic mode VIMF1, as extracted with GiT-EEMD from fMRI data, and *right:* data reconstructed from ERM5. The latter was obtained from EEG data. The comparison concentrates on the late ERP N200.

These results generally comply, in terms of activated regions, with results from an analysis of fMRI data which was taken jointly with our data [7, 62]. This means that these neurons are more active than others which also responded to the contour integration task. In [7, 62], the significant activation differences are highlighted in different regions like occipital, bilateral parietal, temporal and frontal regions (the test has been done using the same p-value, *p* = 0.001, for all). The fact that, comparing both modalities, occasionally different brain regions are involved in contour and non-contour processing renders the comparison suitable for further analysis. Here, for example, with ERM5, the late response *N*200 is pronounced in occipital, temporal and frontal regions, precisely as was found with an fMRI analysis in case of volume intrinsic mode functions (VIMF1, VIMF2, VIMF3 and VIMF4) [7]. Fig 8 presents an illustrative comparison of a saggital view of VIMF1 and ERM5 extracted from the late ERP N200. The VIMFS were extracted by using a new variant of a two dimensional empirical mode decomposition called GiT-BEEMD [63]. Hence, the superior precision in spatial localization of activity blobs corroborates the potential of EEMD/2DEEMD when analyzing functional neuroimages.

## 4 Conclusion

This study investigated the utility of an sLORETA analysis for EEG data from 18 subjects participating in a perceptual learning task. A contour and a non-contour stimulus were presented within the same trial in fast succession, and subjects were asked to indicate their presence *CT* (contour true) or absence *NCT* (non-contour true). The analysis has been performed in two different ways: either using the raw data ERPs or EEMD intrinsic modes called event related modes (ERMs). Note that EEMD has been applied before averaging over trials. Signals have been pooled according to the same clustering scheme of our recent study [4] that divides brain electrodes according to the latencies of the stimulus responses. Early responses are seen in occipital and parietal areas of the brain, while late responses are located in primary visual, medio-temporal and frontal areas. Statistically significant differences between the two stimulus conditions have been seen mainly with ERP components *P*100 and *N*200. The previous study [4] showed that *ERM*5 exhibits very pronounced differences between contour and non-contour stimulus responses, hence only *ERM*5 has been used in the analysis.

As obtained from *ERM*5, earlier differences (before 210*ms*) in source activity between contour and non-contour occurred mainly in occipito-parietal areas, were lateralized to the right hemisphere and showed higher power in the non-contour compared to the contour condition. Later differences (200 − 260*ms*) occurred also in primary visual areas, in both hemispheres and with higher power in the contour compared to the non-contour condition. The latter result fits well with the view that contour integration relies on a top-down flow of information from higher visual areas with large receptive fields into primary visual cortex. The feedback would enhance activity of neurons coding Gabor stimuli at relevant locations and so favor their integration [64, 65]. The former result is partly unexpected in that lower source activity was for contours compared to non-contours. It is possible that the difference reflects the reduced effort of maintaining grouped compared to ungrouped visual input in working memory [66, 67]. In any way, the fact that the difference showed up in right hemisphere complies with the previous finding that contour grouping is a right-lateralized brain function [68].

The results of this study which focuses on identifying related sources of neuronal activation clearly via inverse modeling of EEG data were extremely well matched with the ones in [4] that discussed the forward problem on the same data. Results showed that EEMD method allows to extract components, i.e *ERM*5 which present clearer spatio-temporal differences between the two stimulus responses, *CT* and *NCT* compared to the ERPs of the original signals.
